# Barriers to the dissemination of four harm reduction strategies: a survey of addiction treatment providers in Ontario

**DOI:** 10.1186/1477-7517-3-35

**Published:** 2006-12-14

**Authors:** Karen L Hobden, John A Cunningham

**Affiliations:** 1 Centre for Addiction and Mental Health, Toronto, Ontario, Canada; 2 Centre for Addiction and Mental Health and Departments of Psychology and of Public Health Services, University of Toronto, Toronto, Ontario, Canada

## Abstract

A sample of service providers at addictions agencies' in Ontario were interviewed by telephone to assess attitudes toward, anticipated internal and external barriers to implementing, and expected benefits of four harm reduction strategies: needle exchange, moderate drinking goals, methadone treatment, and provision of free condoms to clients. Respondents were also asked to define harm reduction, list its most important elements, and describe what they find most troubling and most appealing about harm reduction. Attitudes toward harm reduction in general and the services provided at each agency were also assessed. Results indicated that the service providers surveyed had positive attitudes toward each of the four harm reduction strategies and harm reduction in general, and the majority of respondents were aware of the benefits associated with each strategy. Almost all of the agencies surveyed allowed for moderate drinking outcomes in the treatment of alcohol problems, and most agencies provided free condoms to clients. In terms of barriers, anticipated negative community reaction to needle exchange, methadone treatment, and free condoms was a major concern for the majority of respondents. Lack of staff, of funding, or anticipated staff resistance were also cited as potential barriers to introducing these strategies. In the case of methadone maintenance, the unavailability of a qualified physician was listed as the primary constraint. Implications for future efforts directed at encouraging the adoption of these strategies and suggestions for future research are discussed.

## Background

Harm reduction has been gaining popularity in North America as an alternative to traditional means of dealing with substance abuse. Research indicates that harm reduction strategies such as needle exchange and methadone maintenance are associated with reductions in: drug use [1], disease [2-4], crime [2,5] unsafe injection behaviors [1,5], drug related deaths [2], and improvements in employment and interpersonal relationships among IV drug users [5].

Heather [6] suggested that strong empirical evidence demonstrating the effectiveness of harm reduction is necessary to promote its acceptance. Despite the evidence, however, efforts to implement harm reduction strategies have met with resistance from some health care professionals [7-9], especially when dealing with individuals who are considered dependent on rather than just abusing drugs or alcohol [10]. Reasons for this resistance are varied and multifaceted. One difficulty may be the lack of consensus regarding what harm reduction is, exactly. Harm reduction can be defined as any effort that attempts to minimize the negative consequences associated with substance use (either to the individual, their families, their communities, or society as a whole) without requiring the cessation of such use [5,6,10-13]. It is a set of principles that guides the treatment of alcohol and drug problems, as well as the development of public policy relating to drug and alcohol use and is pragmatic, non-judgemental, and client-centered [12,14]. It provides an alternative to the moralistic and medical models of drug and alcohol treatment, acknowledging that some individuals may be unable or unwilling to refrain from use [12,14]. Some authors maintain that safe, controlled substance use is the ultimate goal of harm reduction [5,10], whereas others argue that abstinence is a preferable goal [15,16]. In applying harm reduction to psychotherapy, Denning [11] and Talarsky [17] have suggested that therapeutic success be defined not in terms of amount of drug used, but as any behavior that results in a reduction in drug related harm. Denning [11] has also argued that treatment programs that require abstinence for entry and only allow abstinence as a treatment goal are, in themselves, harmful because they create barriers to treatment for many individuals who might otherwise be helped.

There is some evidence to suggest that attitudes toward harm reduction among professionals in the addictions field may vary as a function of the specific harm reduction strategy employed and the type of service provided. For example, attitudes toward needle exchange were found to be favorable among physicians who treat addictions in Rhode Island [18] and addiction treatment providers in Ontario, Canada [7,19]. In contrast, in their survey of attitudes toward moderate drinking goals among addiction treatment providers in the United States, Rosenberg and Davis [8] found that approximately 75% of reporting agencies considered nonabstinance an unacceptable treatment goal. However, acceptance of moderate drinking goals varied according to type of agency. Approximately one-half of outpatient treatment agencies considered moderate drinking acceptable for some clients. Similar results were reported in Rush and Ogborne's [20] survey of treatment facilities in Ontario and Brocha's [21] survey of private treatment facilities in Quebec. In a nationwide survey of alcohol treatment facilities in Canada, Rosenberg, Devine, and Rothrock [9] found that 62% of outpatient treatment facilities favored moderate drinking goals as a treatment outcome compared to 43% of mixed inpatient/outpatient agencies, 28% of inpatient/detoxification/correctional facilities, and 18% halfway houses.

Ogborne and Birchmore-Timney [7] assessed support for three harm reduction strategies among front line staff in addictions treatment agencies in Ontario: nonabstinence goals in the treatment of alcohol and drug abuse, needle exchange, and methadone maintenance. Results indicated that the staff at outpatient and assessment/referral centers had more favorable attitudes toward harm reduction strategies than those in other types of agencies (e.g. detoxification, and short and long tern residential). Most workers in all types of agencies indicated that they would consider moderate nonabstinent goals for some clients. Needle exchange was acceptable to a majority of workers in all agencies types. There was little acceptance for methadone treatment, with the exceptions of outpatient and assessment/referral staff (the majority of whom were supportive). Similarly, in their survey of addictions treatment providers in Ontario, Ogborne, Wild, Braum, & Newton-Taylor [19] found little support for methadone treatment overall, although support was higher among outpatient and assessment/referral agencies than residential agencies.

According to dissemination researchers, attitudes are only one component in determining whether a new strategy or technology will be adopted [22-24]. Professionals in a given field are not always familiar with the scientific literature describing new methodologies [25-27]. Further, the adoption of any new policy or treatment methodology may be hampered by lack of perceived need, anticipated community resistance, a lack of resources, etc. Rogers [28] identified five stages involved in the processes underlying the adoption of a new technology: knowledge (a basic understanding of the process), persuasion (attitudes), decision (the choice to adopt or reject the innovation), implementation (putting it into practice), and confirmation (evaluating the results of the decision).

The present research was designed to provide an understanding of attitudes toward harm reduction among service providers and the factors influencing agencies' decision to adopt or reject these strategies. Managers and therapists from outpatient and assessment/referral agencies in Ontario were surveyed by telephone. Managers and therapists were chosen as potential respondents because it was assumed at that they would be most aware of their respective agencies' policies and practices regarding the treatment of addictions. Attitudes toward four harm reduction strategies were assessed, as were reasons for accepting or rejecting each of these strategies, internal and external resistance/barriers to introducing them, anticipated benefits of each, reasons for introducing each, and resistance encountered as a result of implementing each. Respondents' own attitudes as well as their estimate of their colleagues' and communities' attitudes toward each strategy were also assessed.

As mentioned previously, there is some disagreement among researchers and theorists concerning the definition of harm reduction. Therefore, respondents were asked to define harm reduction, indicate what elements they consider most important for it, what they find most appealing about it, and what they find most troubling. Finally, their attitude toward harm reduction, in general, was assessed by asking: "how would you feel about helping some alcohol and drug abusers use substances more safety without necessarily reducing the use of these substances?"

## Method

### Materials

A telephone survey explored attitudes toward and use of four harm reduction strategies (needle exchange, moderate drinking goals, methadone treatment, and provision of free condoms to clients). Respondents were asked whether the agency employed the strategy; if not, had they considered it, what the internal and external barriers were, and what benefits would they expect. If the strategy was employed at the agency in question respondents were asked why it was introduced, if there was any internal or external resistance, and, if so, how it was dealt with. Also, each respondent was asked to rate on an 11-point scale (0 = very unfavorable, 10 = very favorable) how they felt about each of the four strategies, how they thought other therapists at their facility felt, and how they thought their community would feel. Five questions dealt with more general attitudes toward harm reduction. Respondents were asked to define it, indicate the most important elements of harm reduction, and state what it is they find most appealing and troubling about harm reduction. Finally, we wanted to get a measure of respondents' overall attitudes toward harm reduction as it is most commonly defined in the literature: as any effort that minimizes the negative consequences associated with substance use without necessarily attempting to reduce or eliminate such use. Therefore, the final question asked respondents to rate on an 11-point scale (0 = very unfavorable, 10 = very favorable) how they would feel about helping some alcohol and drug abusers use substances more safety without necessarily reducing the use of these substances.

### Data collection and survey construction

Data collection took place in two phases. A list of outpatient and assessment/referral agencies in Ontario was obtained from the Drug and Alcohol Registry of Treatment (DART). Each agency was assigned a number. In each of the two phases of data collection, agencies were randomly selected using a random numbers table. Agencies used in the first phase of data collection were exempted from selection in the second phase.

The purpose of the first phase was to develop response categories to the 43 open-ended questions described above. Twenty-two agencies (12 outpatient and 10 assessment/referral) were selected. Managers of each agency were contacted by telephone and asked if they would be willing to participate in a survey of attitudes toward and support for a number of harm reduction strategies. One manager declined. Each manager was asked to suggest a therapist at his/her agency who could also complete the survey. Seventeen therapists were contacted for the survey, the remaining 4 therapists were either unavailable or could not be reached.

All 38 interviews were tape recorded with permission of the respondents. Recordings of each interview were reviewed and responses to each of the open-ended questions were summarized. Commonalities among responses were noted and compiled to form a set of common responses that were used as a basis for constructing response categories for each question.

This semi-structured survey was administered to respondents in the second phase of data collection. The response categories were used as a guideline for coding responses to each question, but questions were still administered in an open-ended format. In cases where respondents' answers did not fit into any of the response categories, the response was coded as "other." Managers from 22 randomly selected agencies (8 outpatient and 14 assessment/referral) were contacted by telephone and details of their responses were noted. Managers from three agencies declined. All managers were asked to suggest a therapist from their agency who could also be surveyed. Ten therapists were contacted for the survey. The remaining nine therapists were either unavailable or could not be reached.

## Results

Managers' and therapists' open-ended responses from the first phase of data collection were recoded into the response categories used in the second phase. Responses from both phases of data collection were combined for analysis. Also, a comparison of means indicated that there were no differences between therapists and managers responses. Therefore, results from all 67 respondents (40 managers and 27 therapists) were aggregated and summary statistics were calculated for each item on the survey. For those items asking whether an agency employed or had considered introducing a program, only managers' responses are reported. We assumed that agency managers would be responsible for making policy decision regarding treatment and would most likely reflect agency policy.

### Needle exchange

Responses to items concerning needle exchange are presented in Table 1. Of the agencies surveyed, 12.5% had a needle exchange program. Of these agencies, four of the eight respondents indicated it was introduced to reduce the spread of HIV and other STDs. Four respondents indicated that some community resistance had been encountered. Of those agencies not using needle exchange, 34.0% had considered it. Reasons for not implementing such a program included: little or no perceived demand (19.0%), the service was already provided locally (19.0%), and the agency was considering it at that time (42.9%). Anticipated internal obstacles to needle exchange included: little or no perceived demand (22.0%), lack of staff (13.6%), and lack of funding (11.9%).

**Table 1:** Frequencies of Responses to Questions on Needle Exchange

**Item**	**N**	**%**
**Agencies currently offering needle exchange (n = 40 agencies)**	5	12.5
**Agencies that considered offering it**	12	34

**Considered it, but decided against it because (n = 21 respondents)**		
Little/no perceived need/demand	4	19.0
service already provided locally	4	19.0
staff resistance	1	4.8
anticipated community opposition	3	14.3
Lack of funding	2	9.5
presently being considered	2	9.5
Don't know	9	42.9
Other	3	14.3

**In agencies not offering needle exchange (n = 59 respondents)**		
**Intra-agency obstacles**		
Little/no perceived need/demand	12	22.0
Lack of medical staff	5	8.5
Lack of funding	7	11.9
service already provided locally	6	10.2
staff resistance	3	5.1
contravenes agency's policy/philosophy	2	3.4
outside mandate/not a medical facility	5	8.5
Lack of staff	8	13.6
Don't know	1	1.7
Other	27	45.8
None	8	13.6
		
**Extra-agency obstacles**		
community resistance/opposition	31	52.5
resistance/opposition from other agencies	3	5.1
Lack of local political support	3	5.1
funding	2	3.4
it would be seen as a duplication of services	1	1.7
May be seen as promoting drug use.	12	20.3
Don't know	2	3.4
Other	6	10.2
None	8	13.6
		
**Expected benefits of needle exchange**		
None	3	5.1
reduction in HIV/STDs	35	59.3
might encourage IV drug users to seek counseling	17	28.8
community safety	7	11.9
greater accessibility/convenience for IV drug users	7	11.9
fallows for greater openness about drug use	3	5.1
Don't know	3	5.1
Other	25	42.4

**For agencies that offer needle exchange (n = 8)**		
**Reasons for introducing it**		
Reduce the spread of HIV/STDs	4	50.0
urged to by AIDS committee/Ministry/other agencies	1	12.5
funding was made available	1	12.5
Other	4	50.0
		
**Resistance encountered**		
None	5	62.5
From the staff	1	12.5
From the Board	1	12.5
From the general community	4	50.0
		
**How was it dealt with?**		
negotiation/conciliation	1	12.5
through education/information	1	12.5
Don't know	1	12.5
Other	1	12.5

In terms of external barriers to needle exchange, most respondents were concerned about community resistance (52.5%) and some felt that a needle exchange program would be seen as promoting drug use (20.3%). When asked about expected benefits, most respondents recognized that needle exchange would reduce the spread of HIV and other STDs (59.3%) and many believed it would encourage IV drug users to seek counselling (28.8%).

### Moderate drinking goals

Responses to items concerning moderate drinking goals are presented in Table 2. Ninety-five percent of agencies surveyed allowed for moderate drinking outcomes in the treatment of alcohol problems. The two agencies that allowed only abstinent outcomes had considered moderation goals. Most respondents indicated that moderate drinking goals were introduced due to client demand (40.3%) or because it was appropriate for some clients (38.7%). Some respondents indicated that for certain clients abstinence was an unrealistic goal (17.7%). When queried as to what, if any, resistance had been encountered, 21% of respondents indicated they had encountered resistance from other agencies, 21% from the AA community, and 11.3% from the staff. Typically this was dealt with through education/information (57.1%).

**Table 2:** Frequencies of Responses to Questions on Moderate Drinking Goals

**Item**	**N**	**%**
**Agencies currently offering moderate drinking goals (n = 40 agencies)**	38	95.0
**Agencies that considered offering moderate drinking goals**	1	2.5

**Considered it, but decided against it because (n = 2 respondents)**		
staff resistance	1	50.0
Other	1	50.0

**For agencies not offering moderate drinking goals (n = 5 respondents)**		
**Intra-agency obstacles**		
not appropriate for their clients	2	40.0
staff resistance	1	20.0
Other	1	20.0
		
**Extra-agency obstacles**		
community resistance/opposition	1	20.0
Other	1	20.0
None	1	20.0
		
**Expected benefits of offering moderate drinking goals**		
some clients find it more appealing than abstinence goals	1	20.0
None	1	20.0

**For agencies that offer moderate drinking goals (n = 62 respondents)**		
**Reasons for introducing it**		
its appropriate for some clients	24	38.7
abstinence is an unrealistic goal for some clients	11	17.7
client demand	25	40.3
empirical evidence supports it	9	14.5
political pressure from outside the agency	1	1.8
harm reduction	7	11.3
Don't know	4	6.5
Other	30	48.4
		
**Resistance encountered**		
a) none	27	43.5
b) from the staff	7	11.3
c) from the Board	5	8.1
d) from the general community	11	17.7
e) from the AA community	13	21.0
f) from other addiction agencies	13	21.0
g) don't know	1	1.6
h) other	6	9.7
		
**How was it dealt with? (n = 35)**		
a) ignored it	3	8.6
b) gave people time to accept it	7	20.0
c) through education/information	20	57.1
d) ran an active PR campaign	1	2.9
e) don't know	1	2.9
f) other	13	37.1

### Methadone maintenance

Only 10% of agencies surveyed had a methadone maintenance program (see Table 3). Of those agencies without a methadone program, 44% had considered implementing one. The most frequently cited reason for not introducing methadone was the unavailability of a physician to administer it (42.9%). Anticipated internal barriers included: the unavailability of a physician (32.3%), little or no perceived need (27.4%), lack of staff (17.7%), lack of funding (11.3%), and staff resistance (11.3%). Some respondents felt that a methadone program would be inappropriate at their agency because they were not a medical facility (11.3%). When asked about obstacles external to the agency, most respondents expressed concern about community resistance (59.7%). In terms of expected benefits, many respondents indicated that methadone treatment improves health and reduces disease in IV drug users (33.9%), is an effective means of getting heroin addicts off heroin (29.0%), results in decreased criminal activity (25.8%), and gives IV drug users access to counselling (12.9%).

**Table 3:** Frequencies of Responses to Questions on Methadone Treatment

**Item**	**N**	**%**
**Agencies that have it (n = 40)**	4	10.0
**Agencies that have considered it (n = 40)**	16	44.0
		
**Considered, but not implemented because (n = 21 respondents)**		
Little/no perceived need	6	28.6
unavailability of a physician willing/able to dispense it	9	42.9
anticipated negative reaction from clientele	1	4.8
Lack of facilities	1	4.8
Lack of funding	2	9.5
prospect of setting up program too daunting	2	9.5
presently being considered	3	14.3
Other	4	19.0
		
**Agencies that do not have a methadone program (n = 62 respondents)**		
**Intra-agency obstacles**		
Little/no perceived need	17	27.4
unavailability of a physician willing/able to dispense it	20	32.3
Lack of facilities	2	3.2
Lack of funding	7	11.3
not a medical facility/outside agency's mandate	7	11.3
anticipated resistance from the staff	7	11.3
May be some resistance from the Board	5	8.1
Don't know	2	3.2
Other	17	27.4
None	4	6.5
Lack of staff	11	17.7
		
**Extra-agency obstacles**		
None	12	19.4
community resistance	37	59.7
resistance from other agencies	2	3.2
service already provided locally	2	3.2
Don't know	4	6.5
Other	11	17.7
		
**Expected benefits**		
None	3	4.8
improved health/disease reduction	21	33.9
enables clients to be more productive	10	16.1
decreased criminal activity	16	25.8
gives drug users access to counseling	8	12.9
enables addicts to get off heroin	18	29.0
Don't know	4	6.5
Other	18	29.0
		
**For agencies who offer methadone (n = 5 respondents)**		
**Reasons for introducing it.**		
		
perceived need	2	40.0
urged to do so by the Ministry of Health/other agencies	1	20.0
Other	3	60.0
		
Resistance encountered		
None	2	40.0
From the staff	2	40.0
From the community	2	40.0
		
**How was it dealt with?**		
through education/information	2	40.0

### Provision of free condoms to clients

Responses to the survey indicated that most agencies (67.5%) make free condoms available to their clients (see Table 4). Of the 13 agencies where free condoms were not provided, four had considered making them available. Results indicated little concern regarding internal obstacles to providing condoms, but many respondents expressed concerns about negative community reactions (66.7%). Most respondents acknowledged that condoms are an effective means of reducing transmission of HIV and other STDs (81.0%). Respondents at agencies that provide free condoms indicated that the measure was introduced primarily as a means of reducing HIV/STD transmission (58.7%). Interestingly, 75.5% of these respondents indicated that no resistance was encountered to the introduction of this measure.

**Table 4:** Provision of free condoms

**Item**	**N**	**%**
**Agencies that offer them (n = 40)**	27	67.5
**Agencies that have considered it (n = 40)**	4	10.0
		

**Considered, but not implemented because (n = 5 respondents)**		
resistance from the staff	1	20.0
resistance from the Board	1	20.0
felt it was inappropriate for clientele	1	20.0
it contravenes agency policy/philosophy	1	20.0
Don't know	1	20.0
Other	2	40.0
		
**Agencies that do provide free condoms (n = 21 respondents)**		
**Intra-agency obstacles**		
None	5	23.8
staff resistance	3	14.3
resistance from the Board	2	9.5
concerns about negative community reactions	2	9.5
Lack of funding	3	14.3
Other	10	47.6
		
**Extra-agency obstacles**		
None	5	23.8
community resistance	14	66.7
Don't know	1	4.8
Other	3	14.3
		
**Expected benefits**		
reduction in STDs/AIDS	17	81.0
reduction in unwanted pregnancy	10	47.6
opportunity to provide information/education	6	28.6
Other	3	14.3
		
**For agencies that offer free condoms (n = 46)**		
**Reasons for offering them**		
to reduce HIV/STDs	27	58.7
to reduce unwanted pregnancy	9	19.6
to provide information/education	7	15.2
funding was made available	2	4.3
urged to do so by the Ministry of Health/other agencies	3	6.5
Don't know	7	15.2
Other	20	43.5
		
**Resistance encountered**		
None	34	73.9
From the staff	3	6.5
From the community	5	10.9
From other agencies	1	2.2
Don't know	2	4.5
Other	2	4.5
		
**How was it dealt with? (n = 11)**		
ignored it	1	9.1
gave people time to accept it	2	18.2
through education/information	4	36.4
Other	2	18.2

### Attitudes toward the four harm reduction strategies

In order to determine whether respondents' attitudes varied by type of agency (outpatient versus assessment/referral), separate MANOVAs were performed on respondents' assessments of their own, their colleagues', and their communities' attitudes toward each of the four harm reduction strategies. Significant univariate ANOVAs were examined subsequently. The only significant difference found by agency type was in respondents' perceptions of their communities' feelings about nonabstinence as a treatment goal. Respondents from outpatient facilities perceived that their community would be significantly less accepting of moderate drinking outcomes (x = 5.76) than their counterparts in assessment/referral agencies (x = 6.75), F(1, 50) = 4.79. No other differences by agency type were found.

Repeated measures analysis of variance (ANOVA) and paired t-tests were used to compare respondents' attitudes toward each of the four harm reduction strategies to their estimates of their colleagues' and communities' attitudes. Results are presented in Table 5. Respondents reported positive attitudes toward needle exchange (x = 9.03), but felt their colleagues (*x *= 8.43), and their community would be less favorable (*x *= 4.90), *t*(59) = 4.87, *p *< .01 and *t*(54) = 12.72, respectively, *F*(2,48) = 91.31, *p *< .01. Mean attitudes toward moderate drinking goals were also positive (*x *= 9.04), but respondents expected their colleagues (x = 8.60), and community would be comparatively less favorable (x = 5.97), *t*(61) = 3.10, *p *< .01 and *t*(53) = 13.10, respectively, *F*(2,49) = 102.44, *p *< .01. Respondents were accepting of methadone treatment (x = 8.19), but felt that their colleagues (x = 7.81), and community (x = 4.79) held comparatively less favorable attitudes, *t*(54) = 3.08, *p *< .01 and *t*(49) = 11.02, respectively, *F*(2,42) = 54.24, *p *< .001. Finally, respondents' attitudes toward the provision of free condoms to clients were favorable (*x *= 9.46), as were estimates of their colleagues' attitudes (*x *= 9.39). However, respondents' anticipated that members of their community would be comparably less favorable (*x *= 6.51), *t*(56) = 11.64, *p *< .01, *F*(2,51) = 57.04, *p *< .001.

**Table 5:** Mean responses to attitude measures (n = 67)

**Item**	**Mean**
*How do you feel about providing clean needles to drug users?*	*9.03*
How do you think other therapists at your agency feel (about needle exchange)?	8.42
How do you think (needle exchange) would be viewed by your community?	4.90

How do you feel about nonabstinence as a treatment goal for some clients?	9.04
How do you think other therapists at your agency feel (about nonabstinence)?	8.60
How do you think (nonabstinence) would be viewed by your community?	5.97

How do you feel about offering methadone treatment as a treatment option?	8.19
How do you think other therapists at your facility feel (about methadone)?	7.81
How do you think methadone treatment would be viewed by your community?	4.79

How do you feel about providing free condoms to clients in treatment facilities?	9.46
How do you think other therapists at your facility feel (about providing free condoms)?	9.39
How do you think providing free condoms would be viewed by your community?	6.51

Scores range from 0 to 10 with higher scores indicating more positive attitudes.

### Harm reduction

Frequency and mean responses to the five more general attitude questions concerning harm reduction are presented in Table 6.

**Table 6:** General harm reduction questions (n = 67)

**Item**	**N**	**%**
**Definitions of harm reduction**		
reducing harm from substance use I incurred by the individual by reducing or eliminating the use of that substance	3	4.5
reducing the harm from substance use incurred by the individual and reducing their use of that substance	6	9.0
reducing the harm from substance use incurred by the individual without necessarily reducing their use of that substance	16	23.9
reducing the harm associated with substance use to the community or society as a whole	8	11.9
don't know	3	4.5
Other	36	53.7
reducing the harm associated with substance use	7	10.4
		
**Most important elements of harm reduction**		
disease reduction	4	6.0
empowering the client	7	10.4
improving the quality of life of client	4	6.0
reducing negative consequences associated with drug/alcohol use	1	1.5
flexibility/options	7	10.4
education/awareness on the part of the client	13	19.4
education/awareness on the part of the community	8	11.9
client choice	11	16.4
empathy	5	7.5
accurate assessment	2	3.0
don't know	3	4.5
Other	40	59.7
		
**Most appealing aspects of harm reduction**		
disease reduction	6	9.0
reduced health costs	2	3.0
May provide a gateway/bridge into treatment	6	9.0
it's more palatable to clients than abstinence	6	9.0
client choice	16	23.9
it's nonjudgmental	13	19.4
it's client centered	14	20.9
it's appropriate for some clients	3	4.5
it's pragmatic/practical	9	13.4
it provides flexibility/options	7	10.4
it empowers the client	8	11.9
don't know	1	1.5
Other	24	35.8
		
**Most troubling aspects of harm reduction**		
it's not appropriate for or in the best interest of all clients	14	20.9
it's often misunderstood and misapplied	14	20.9
it creates moral and ethical dilemmas for the community	2	3.0
it creates moral and ethical dilemmas for the therapist	8	11.9
negative public attitudes	4	6.0
it's difficult to use illicit drugs safely	2	3.0
abstinence is better	7	10.4
harm reduction should be accompanied by counseling	2	3.0
don't know	2	3.0
Other	38	56.7
		

	***Mean***	
On a scale from 0 to 10 with 0 = "not at all favorable" and 10 = "extremely favorable," how would you feel about helping some alcohol/drug abusers use substances more safely without necessarily trying to reduce their use of these substances?	6.51	

#### Definition

Results indicated that there was little agreement concerning what harm reduction actually is. Most responses (53.7%) fell into the "other" category (e.g., "It's making wise personal choices based on available information," "Awareness and knowledge," "An attitude set"). Only 23.9% of respondents defined harm reduction as reducing the harm associated with substance use without necessary reducing the use of that substance.

#### Most important elements, appealing features, and troubling aspects

Features most commonly cited as important elements of harm reduction were: increasing client awareness/education (19.4%) and client choice (16.4%). Features listed as most appealing aspects of harm reduction included such things as: it gives clients choice (23.9%), it's client-centred (20.9%), and it's non-judgemental (19.4%). The most troubling aspect of harm reduction given was that it is not in the best interest of all clients (20.9%) and is often misunderstood and/or misapplied (20.9%).

#### Overall attitude toward harm reduction

Respondents were asked to rate how they would feel about helping some alcohol and drug abusers use substances more safety without necessarily reducing the use of these substances. The mean response to this question was positive (x = 8.49), suggesting service providers have favorable attitudes toward harm reduction in general. A one-way ANOVA on overall attitudes toward harm reduction in general failed to find significant differences by agency type.

## Discussion

Responses to questions concerning needle exchange indicated that only a small percentage of agencies surveyed offered this service. Almost half of those agencies not offering a needle exchange program were considering introducing one at the time of this survey. When asked about expected benefits, the majority of respondents recognized that needle exchange is an effective way of reducing the spread of HIV and other STDs. The most commonly cited barrier was anticipated community resistance. More than half the respondents indicated they would expect a negative response from their local communities. In addition, for some agencies, lack of staff and funding were also a concern.

Almost all agencies surveyed offered moderate drinking goals as a treatment option for some individuals with alcohol problems. The most frequently cited reasons for introducing such goals were client demand and the belief that nonabstinence is an appropriate treatment goal for certain clients. Some respondents indicated that they had encountered resistance to moderate drinking goals from the AA community and other agencies, but that this was dealt with effectively through education and dialogue.

Only a few agencies surveyed offered a methadone treatment program, but close to half had considered implementing one. The most commonly cited reason for not offering methadone was the inability to find a physician qualified and/or willing to administer it. Approximately one-third of respondents indicated that there was little or no perceived need for such a program in their community. The majority of respondents felt that their communities would respond negatively to the introduction of a methadone program. Some respondents were concerned about lack of staff and staff resistance. Approximately one-third of respondents recognized that methadone treatment reduces the incidence of disease and improves the health of IV drug users, while providing an effective means of treating opiate addiction. Many respondents also acknowledged that methadone treatment programs contribute to a reduction in criminal activity.

Responses concerning provision of free condoms to clients indicated that approximately two-thirds of agencies surveyed already offered this service. For those agencies not offering free condoms, concerns about negative community reaction were the most commonly cited barrier, although most of these respondents acknowledged that providing free condoms would be an effective way of reducing the transmission of HIV and other STDs. For those agencies providing free condoms, the majority of respondents indicated that the service was implemented as a means of reducing the spread of HIV/STDs. Interestingly, 75% of these agencies reported no community resistance.

Results from our attitudinal measures parallel those of Ogborne et al. [19] and Rush and Ogborne [20]. Respondents held positive attitudes toward needle exchange, moderate drinking goals, methadone maintenance, and provision of free condoms. The attitudes of treatment providers in Canada stand in stark contrast to those in the United States, where attitudes towards moderate drinking outcomes tend to be negative [8]. The exact reasons for this difference is a topic for future research, but may be related to differences in values and attitudes between the two nations. A comparison of attitudes toward alcohol and drug use across several nations [29] found that respondents in the US had more of a moralistic or "temperance mentality" towards alcohol and drug use than those in Canada.

One interesting finding was the disparity between respondents' self-reported attitudes toward each of the harm reduction strategies and their estimates of their colleagues' and communities' attitudes. For three of the four harm reduction strategies respondents assumed both their colleagues and their communities held more negative attitudes than they did. Only in the case of providing free condoms to clients did respondents assume that their colleagues would be equally favorable, but still believed their communities would be significantly less favorable. One wonders at the source of this perception. Perhaps respondents were disowning their own misgivings about these strategies and projecting them onto their colleagues and neighbours. Equally plausible, respondents may have conceptualized themselves as unusually progressive compared to their colleagues and community members. It is unlikely, however, that in the course of our survey we just happened across the one person in each agency who was most in favor of harm reduction. Whatever the reason for these perceptions, greater communication both within agencies and between agencies and communities might help treatment providers recognize that they are not alone in their convictions.

Our results found little agreement among service providers as to what harm reduction is. The majority of definitions given did not fall into any of the five categories we had listed. Further, there was little consensus concerning what constitutes the core elements of harm reduction. This apparent confusion in the field may undermine attempts to promote harm reduction as a distinct paradigm and may complicate efforts to measure attitudes and rate of adoption of these policies.

In terms of Rogers' [29] dissemination model, our findings suggest that the greatest impediments to adoption of these harm reduction strategies in Ontario have to do with issues of implementation, resources, and motivation, rather than with knowledge or attitudes. Factors such as anticipated negative community reactions as well as funding and staffing concerns were cited most often as barriers to implementing these strategies. These findings suggests that future efforts to promote harm reduction need to focus, not on persuasion, but on addressing service providers' specific concerns regarding community reactions and agency resources.

It is possible that responses to this survey were influenced by social desirability concerns. In an effort to counteract such concerns, respondents were assured that all responses would be confidential and that respective agencies would not be cited in any publication of this data. Also, the results of this study are limited to the population sampled (i.e., managers and therapists of outpatient treatment and assessment referral centres in Ontario). One possible direction for future research would be to sample more broadly treatment providers across Canada to see if these results generalize to treatment providers in other provinces. Additionally, it would be interesting to survey public opinion toward harm reduction strategies in either nation in an effort to determine the accuracy of our respondents' expectations that their communities' attitudes would be more negative than their own. Results of such research may help to either allay concerns about negative community reactions on the part of addiction treatment providers or alert them to this potential barrier so that strategies can be developed for dealing with it.

## Authors' contributions

KH conceived of the study, designed the survey, and collected and analysed the data under the supervision of JC.

## Appendix A

### Harm reduction survey

#### Needle exchange

1. Does your agency currently have a needle exchange program?

Y (go to Q7) N (go to Q2.)

2. Has your agency considered implementing a needle exchange program?

Y (go to Q3) N (go to Q4)

3. Why was it not implemented?

a) Little/no perceived need/demand

b) Service already provided locally

c) Staff resistance

d) Anticipated community opposition

e) Anticipated negative client reaction

f) Lack of funding

g) Presently being considered

h) Don't know

I) Other

4. What are the internal barriers to implementing needle exchange?

a) Little/no perceived need/demand

b) Lack of medical staff

c) Lack of funding

d) Service already provided locally

e) Staff resistance

f) Contravenes agency's policy/philosophy

g) Outside mandate/not a medical facility

h) Don't know

I) Other

j) Lack of staff

k) None

5. What are the external barriers?

a) Community resistance/opposition

b) Resistance/opposition from other agencies

c) Lack of local political support

d) Funding

e) It would be seen as a duplication of services

f) May be seen as promoting drug use.

g) Don't know

h) Other

I) None

6. What benefits would you expect from needle exchange

a) None

b) Reduction in HIV/STDs

c) Might encourage IV drug users to seek counselling

d) Community safety

e) Greater accessibility/convenience for IV drug users

f) Allows for greater openness about drug use

g) Don't know

h) Other

Agencies that have needle exchange (Q7–9)

7. Why was it introduced? (Check all that apply)

a) Reduce the spread of HIV/STDs

b) Urged to by AIDS committee/Ministry/Other agencies

c) Funding was made available

d) Don't know

e) Other

8. Was there any internal or external resistance encountered? (Check all that apply)

a) None

b) From the staff

c) From the Board

d) From the general community

e) From the AA community

f) From other addiction agencies

g) Don't know

h) Other

9. How was it dealt with? (Check all that apply)

a) Ignored it

b) Negotiation/conciliation

c) Gave people time to accept it

d) Through education/information

e) Ran an active PR campaign

f) Don't know

g) Other

All agencies (Q10–12)

10. How do you feel about providing clean needles to drug users on a scale of 0 to 10 (where 0 = "not at all favorable" and 10 = "extremely favorable")?

11. Using the same scale, how do you think other therapists at your agency feel about needle exchange (0 = "not at all favorable" and 10 = "extremely favorable")?

12. How do you think needle exchange would be viewed by your community (0 = "not at all favorable" and 10 = "extremely favorable")?

#### Moderate drinking goals

13. Does your agency allow for moderate drinking goals the treatment of alcohol problems? Y(go to Q19) N (go to Q14)

14. Have you considered it? Y (go to Q15) N (go to Q16)

15. Why was it not adopted? (Check all that apply)

a) Not appropriate for their clientele

b) Little/no perceived need/demand

c) The service already provided locally

d) Staff resistance

e) Anticipated opposition from community

f) Anticipated negative client reaction

g) Lack of funding

h) Presently being considered

I) Don't know

j) Other

16. What are the internal barriers to moderate drinking goals? (Check all that apply)

a) Not appropriate for their clientele

b) Staff resistance

c) Resistance from the Board

d) Contravenes agency's policy/philosophy

e) Don't know

f) Other

g) None

17. What are the external barriers? (Check all that apply)

a) Community resistance/opposition

b) Resistance/opposition from other addiction agencies

c) Negative reaction from the AA community

d) Don't know

f) Other

g) None

18. What benefits would you expect from offering moderate drinking goals as a treatment outcome? (Check all that apply)

a) Some clients find it more palatable/appealing than abstinence

b) It is appropriate for some clients

c) Flexibility/options

d) Don't know

e) Other

f) None

For agencies that allow for moderate drinking outcomes (Q19–22)

19. Why were moderate drinking goals adopted as a treatment outcome? (Check all that apply)

a) It's appropriate for some clients

b) Abstinence unrealistic goal for some clients

c) Client demand

d) Empirical evidence supports it

e) Political pressure from outside the agency

f) Harm reduction

g) Don't know

h) Other

20. Was there any internal or external resistance encountered? (Check all that apply)

a) None

b) From the staff

c) From the Board

d) From the general community

e) From the AA community

f) From other addiction agencies

g) Don't know

h) Other

21. How was it dealt with?

a) Ignored it

b) Gave people time to accept it

c) Through education/information

d) Ran an active PR campaign

e) Don't know

f) Other

All agencies (Q22–26)

22. How do you feel about nonabstinance as a treatment goal for some clients on a scale of 0 to 10 (where 0 = "not at all favorable" and 10 = "extremely favorable")?

23. Using the same scale, how do you think other therapists at your agency feel (0 = "not at all favorable" and 10 = "extremely favorable")?

24. How do you think nonabstinance goals would be viewed by your community (0 = "not at all favorable" and 10 = "extremely favorable?

25. Agency's policy regarding clients who fail to remain abstinent while in a treatment program

a) Discharged completely

b) Asked to leave for a period of time

c) Allowed to stay in treatment, reasons for Relapse explored

d) Allowed to stay in treatment, goals Reassessed

e) Don't know

f) Other

g) Allowed to stay in treatment/continue to Work with them

26. Type of treatment offered by agency.

a) 12 step/spiritual

b) CBT

c) GSC

d) SRP

e) Motivational interviewing

f) transtheoretical/Stages of Change

g) Systems approach

h) Client-centred/Rogerian

I) Solution-focused

j) Brief intervention

k) Don't know

l) Other

#### Methadone maintenance

27. Does your agency have a methadone maintenance program?

Y (go to Q33) N (go to Q2)

28. Have you considered it? Y (go to Q29) N (go to Q30)

29. Why was it not implemented?

a) Little/no perceived need

b) Unavailability of a physician willing/able to dispense it

c) Anticipated negative reaction from clientele

c) Anticipated negative reaction from staff

d) e) Anticipated negative reaction from Board

e) f) Lack of facilities

f) Lack of funding

g) h) Prospect of setting up

Program too daunting

I) Presently being considered

j) Don't know

k) Other

30. What internal barriers where there to introducing a methadone maintenance program?

a) Little/no perceived need

b) b) Unavailability of a physician willing/able to dispense it

c) Lack of facilities

d) Lack of funding

e) Not a medical facility/outside agency's mandate

f) Anticipated resistance from the staff

g) May be some resistance from the Board

h) May be some resistance from clientele

I) Don't know

j) Other

k) None

l) Lack of staff

31. What external barriers were there?

a) None

b) Community resistance

c) Resistance from other agencies

d) Service already provided locally

e) Don't know

f) Other

32. What benefits would you expect from methadone maintenance?

a) None

b) Improved health/disease reduction

c) Enables clients to be more productive

d) Decreased criminal activity

e) Gives drug users access to counselling

f) Enables addicts to get off heroin

g) Don't know

h) Other

For agencies that offer methadone only (Q33–35)

33. What were your reasons for introducing a methadone maintenance program?

a) Perceived need

b) Funding was made available

c) Urged to do so by the Ministry of Health/Other agencies

d) Don't know

e) Other

34. Did you encounter any internal or external resistance?

a) None

b) From the staff

c) From the Board

d) From the community

e) From other addiction agencies

f) Don't know

g) Other

35. How was it dealt with?

a) Ignored it

b) Gave people time to accept it

c) Through education/information

d) Ran an active PR campaign

e) Don't know

f) Other

All agencies (Q36–38)

36. How do you feel about offering methadone treatment as a treatment option on a scale from 0 to 10 where 0 = "not at all favorable" and 10 = "extremely favorable"?

37. How do you think other therapists at your facility feel about methadone (0 = "not at all favorable" and 10 = "extremely favorable")?

38. How do you think methadone treatment would be viewed by your community (0 = "not at all favorable" and 10 = "extremely favorable")?

#### Provision of free condoms

39. Does your agency provide free condoms to clients?

Y (go to Q45) N (go to Q40)

40. Have you ever considered it? Y (got to Q41) N (go to Q42)

41. Why was it not implemented?

a) Resistance from the staff

b) Resistance from the Board

c) Fear of negative community reaction

d) Felt it was inappropriate for clientele

e) It contravenes agency policy/philosophy

f) Don't know

g) Other

42. What external barriers were there to offering free condoms to clients?

a) None

b) Staff resistance

c) Resistance from the Board

d) Concerns about negative reactions from clients

e) Concerns about negative community reactions

f) Lack of funding

g) Don't know

h) Other

43. What external barriers were there?

a) None

b) Community resistance

c) Opposition from other agencies

d) Don't know

e) Other

44. What benefits would you expect from offering free condoms to clients?

a) Reduction in STDs/AIDS

b) Reduction in unwanted pregnancy

c) Opportunity to provide Information/education

d) Don't know

d) Other

For agencies that offer methadone (Q 45–47)

45. Reasons for offering them

a) To reduce HIV/STDs

b) To reduce unwanted pregnancy

c) To provide information/education

d) Funding was made available

e) Urged to do so by the Ministry Of Health/other agencies

f) Don't know

g) Other

46. Did you encounter any internal or external resistance?

a) None (go to Q47)

b) From the staff

c) From the Board

d) From the community

e) From other agencies

f) Don't know

h) Other

47. How was it dealt with?

a) Ignored it

b) Gave people time to accept it

c) Through education/information

d) Ran an active PR campaign

e) Don't know

f) Other

All agencies (Q48–55)

48. How do you feel about providing free condoms to?

Clients on a scale from 0 to 10 with 0 = "not at all favorable" and 10 = "extremely favorable"?

49. How do you think other therapists at your facility feel about providing free condoms (0 = "not at all favorable" and 10 = "extremely favorable")?

50. How do you think providing free condoms would be viewed by your community 0 = "not at all favorable" and 10 = "extremely favorable"?

#### General harm reduction questions

51. Definitions of harm reduction

a) Reducing harm from substance use incurred by the individual by reducing or eliminating the use of that substance

b) Reducing the harm from substance use incurred by the individual and reducing their use of that substance

c) Reducing the harm from substance use incurred by the individual without necessarily reducing their use of that substance

d) Reducing the harm associated with substance use to the community or society as a whole.

e) Don't know

f) Other

g) Reducing the harm associated with substance use

52. Most important elements of harm reduction

a) Disease reduction

b) Empowering the client

c) Improving the quality of life of client

d) Reducing negative consequences associated with drug/alcohol use

e) Flexibility/options

f) Education/awareness on the part of the client

g) Education/awareness on the part of the community

h) Client choice

i) Empathy

j) Accurate assessment

k) Don't know

l) Other

53. Most appealing aspects of harm reduction.

a) Disease reduction

b) Reduced health costs

c) May provide a gateway/bridge into treatment

d) It's more palatable to clients than abstinence

e) Client choice

f) It's non-judgemental

g) It's client centred

h) It's appropriate for some clients

i) It's pragmatic/practical

j) It provides flexibility/options

k) It empowers the client

l) Don't know

m) Other

54. Most troubling aspects of harm reduction

a) It's not appropriate for or in the best interest of all clients

b) It's often misunderstood and misapplied

c) It creates moral and ethical dilemmas for the community

d) It creates moral and ethical dilemmas for the therapist

e) Negative public attitudes

f) It's difficult to use illicit drugs safely

g) Abstinence is better

h) Harm reduction should be accompanied by counselling

i) Don't know

j) Other

55. On a scale from 0 to 10 with 0 = "not at all favorable" and 10 = "extremely favorable," how would you feel about helping some alcohol/drug abusers use substances more safely without necessarily trying to reduce their use of these substances?
